# The Microscope

**Published:** 1877-07

**Authors:** 


*The Microscope. A Manual of Microscopy, and Compendium of the Microscopic Sciences, Micro-Mineralogy, Micro-Chemistry, Biology, Histology and Pathological Histology.*—Third Edition. Re-written and greatly enlarged, with Two Hundred and Five Illustrations. By J. H. WYETH A. M., M. D., Professor of Microscopy and Biology, in the Medical College of the Pacific, San Francisco. Philadelphia: Lindsay & Blakiston. 8vo. pp. 259.

The author dedicated this volume to the San Francisco Microscopical Society, and its character is such as adapts it to the general student in microscopy. The first chapters are upon the construction and use of the instrument, and the others present a *resume* of the points of interest in the various departments of microscopical study. The numerous well executed, plain and colored plates reproduced here from other authors, add much to the value of the work. It is worthy of recommendation for the brief and interesting manner with which subjects are treated and facts brought to notice; for the suitable directions given for collection and preparation of specimens; and for comprehensive views presented in tables of general classification and lists, such as those of fungi, diatoms, infusoria etc. The portion which presents animal histology is comprehensive, concise and interesting. It is a work of value to the general student. W.

